# Asthma: effect of excess short-acting β_2_-agonist (SABA) inhaler prescriptions on healthcare resource utilisation

**DOI:** 10.3399/bjgp24X739089

**Published:** 2024-07-26

**Authors:** Mark L Levy, Toby GD Capstick, Thomas Antalffy

**Affiliations:** Kenton Bridge Medical Centre, Harrow.; Respiratory Medicine, Leeds Teaching Hospitals NHS Trust, Leeds.; Smart Respiratory Ltd, London.

## Introduction

Asthma management is changing, and the preferred option (track 1) recommended by the Global Initiative for Asthma (GINA) is that all people with asthma should be prescribed an inhaled corticosteroid either as needed in mild asthma or regularly for those with more severe asthma.^1^ Short acting β_2_-agonist bronchodilators (SABAs) certainly have an essential role in the management of acute asthma attacks, but their regular use in the chronic management of asthma has recently been highlighted as having potentially dangerous adverse clinical effects, especially when used without inhaled corticosteroids.^1^^,^^2^ The association between excess prescriptions of SABAs and increased asthma attacks, healthcare utilisation, and deaths has been known for decades,^3^^,^^4^ and illustrated so clearly in the SABINA studies^5^^,^^6^ and the UK National Review of Asthma Deaths (NRAD).^7^ Furthermore, it is 30 years since Suissa *et al*^3^^,^^4^ demonstrated the association between asthma death and one or more SABA inhaler prescriptions a month with a concomitant reduction in mortality when inhaled corticosteroids (ICS) were used.

In 2022/2023, NHS England published a financial incentive scheme to reward Primary Care Networks (PCNs) for reducing the percentage of patients on asthma registers who received six or more SABA inhaler prescriptions over the previous 12 months;^8^ the scheme was later replaced with one focused on access to care. Excess SABA prescribing is now defined as the prescription of three or more SABA inhalers in 12 months, because this level is associated with more frequent and more severe asthma attacks, as well as mortality.^5^^,^^6^

It is clear to us that continuing with an asthma management strategy where SABA remains an integral component of the treatment plan will continue to risk avoidable asthma attacks, hospital admissions, and asthma deaths. We have recently recommended a change in UK asthma management, utilising the anti-inflammatory reliever (AIR) therapy approach to replace SABA reliever prescribing with inhaled corticosteroid inhalers in combination with formoterol, a quick-acting, long-acting bronchodilator.^9^ In the UK, NHS primary and secondary care budgets are not interlinked. As a result, there are concerns that managers and commissioners focus on primary care prescribing costs in isolation, with less consideration of the impact on future hospital costs. Consequently, changes in unscheduled healthcare utilisation and associated healthcare resource hospital costs are not analysed in association with those incurred because of primary care prescribing. In this analysis, we try to explain the financial costs associated with excess SABA prescription, a surrogate measure of poorly controlled asthma, and usage based on published healthcare utilisation data.^10^^,^^11^

## Healthcare resource utilisation associated with SABA inhaler prescriptions

Attar-Zadeh *et al*^10^ utilised data retrospectively from 186 061 patients aged ≥12 years who were diagnosed with asthma, from a large UK database, to analyse healthcare resource utilisation (HCRU) and medication costs related to low and high numbers of prescriptions of SABA inhalers. Low and high SABA prescriptions were defined in this paper as 1 to 2 and ≥3 inhaler canisters per year respectively. The authors concluded that high versus low SABA prescriptions were associated with higher HCRU costs. Also, there were higher overall HCRU costs across all treatment steps in the high-SABA compared with the low-SABA group.^10^ The total cost for the high-SABA group (*n* = 94 544) was £7 910 112 (that is, £83.67 per patient) per year, compared with a total cost for the low-SABA group (*n* = 91 517) of £3 402 384 (£37.18 per patient) per year. The HCRU costs were greater in the high-SABA users because of higher non-exacerbation-related HCRU (primary care without oral corticosteroid, and outpatient visits) and exacerbation-related HCRU (primary care with oral corticosteroid, Accident and Emergency [A&E] attendance, and admissions to hospital). Annual hospital admissions were 3.3 times higher in the high-compared with low-SABA groups (1271 versus 391) (see Supplementary Table S3 in Attar-Zadeh *et al*).^10^ For this 3.3 times increased rate of admissions to hospital, the overall increased HCRU went up by a factor of 2.25 (£83.67/£37.18). These data enabled a calculation of an ‘HCRU cost ratio’ of 1271/391, that is, 3.3, and demonstrate that increasing from 1 unit of HCRU to 3.3 units of HCRU in this population drove the overall HCRU costs from £37.18 per patient per year to £83.67 in the low-versus high-SABA groups respectively.

In a smaller study, a multivariate analysis of prescribing in 139 practices in London, Hull *et al*^11^ concluded that patients prescribed more than three SABA inhalers a year were at increased risk of admission to hospital due to asthma. These authors classified SABA prescriptions per year in three groups (1–3 [*n* = 9451], 4–12 [*n* = 10 285], and >12 SABA [*n* = 1148] prescriptions per year). In this study the crude inpatient episode (hospital admission) rates for asthma attacks in 20 884 cases who were prescribed insufficient inhaled corticosteroids (either alone or in combination with long-acting bronchodilators) were 5.8 times greater in the highest SABA users compared with the lowest users (1.29, 2.32, and 7.49 in the 1–3, 4–12, and >12 SABA groups respectively). Assuming that the overall HCRU costs increase linearly with the hospital admission rates, a crude estimated overall HCRU cost per year in those prescribed >12 SABA inhalers in this study^11^ may be extrapolated using the ‘hospital admission ratio’ calculated for the Attar-Zadeh study,^10^ to users of >12 SABAs per year:

37.17+([{£83.67-£37.18}/{3.3-1}]*[5.8-1])=£134.19

Since the numbers of hospital admissions in the Hull *et al* study were 5.8 times greater in the highest SABA users,^11^ a proportional increase in overall HCRU cost of £134.19 may be assumed.

**Figure 1.**
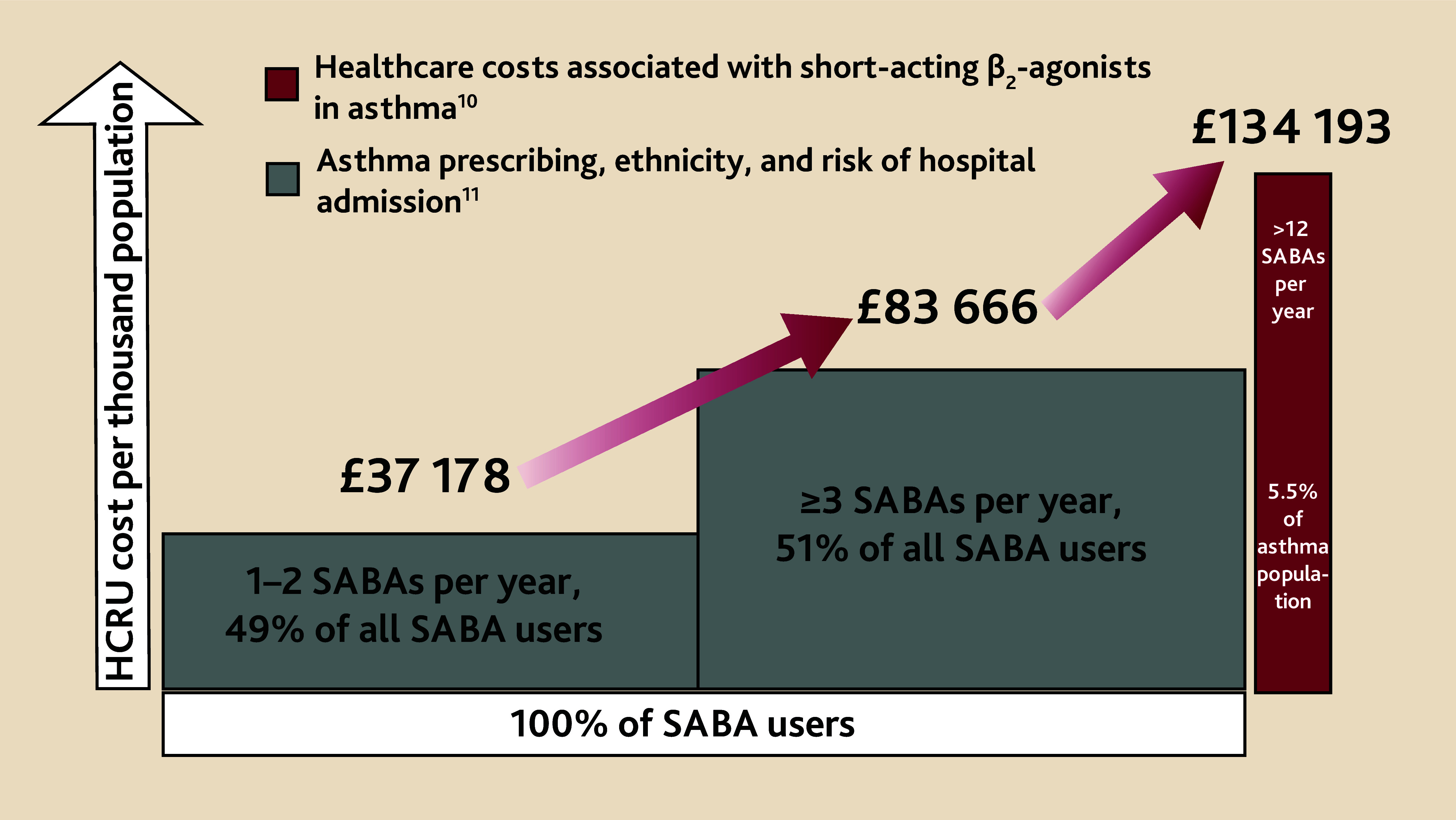
Hidden healthcare resource costs associated with numbers of SABA prescriptions. Annual prescribing costs for 1–2 (low) and ≥3 (high) SABA prescriptions in one study population (*n* = 186 061) with a hospital admission ratio of 3.3 for these two groups.^10^ This ratio facilitated extrapolation of costs for a second study population (*n* = 20 884) with a hospital admission ratio of 5.8 in those prescribed ≤3 and >12 SABA inhalers per year.^11^ HCRU = healthcare resource utilisation. SABA = short-acting β_2_-agonist bronchodilators.

Figure 1 summarises the data from the two studies above^10^^,^^11^ and demonstrates the marked increase in annual unscheduled healthcare utilisation costs due to hospital admission for asthma attacks related to associated excess SABA prescriptions. The overall calculated HCRU costs are £37 178, £83 666, and £134 193 per 1000 patients with low (1–2), high (3–12), and very high (>12) use of SABA inhalers per year.

We have highlighted the large increase in HCRU costs associated with people prescribed excess quantities of SABA inhalers, and who should therefore be targeted for urgent review. This information is clearly helpful for clinicians who could reduce the risk of future attacks with a structured asthma review (https://www.ipcrg.org/dth2) and optimisation of care. It is also useful for those responsible for planning and implementing asthma care as well as those responsible for cost-effective optimisation use of medication in primary care. Furthermore, these data provide justification for implementing the anti-inflammatory reliever therapy approach utilising a two-in-one inhaler that delivers an anti-inflammatory inhaled corticosteroid-formoterol reliever, where formoterol is a quick-acting, long-acting bronchodilator.^9^ Implementing a SABA-free prescribing strategy that prevents asthma exacerbations will reduce asthma admissions to hospital^9^ and is likely to reduce overall HCRU costs, even at the expense of prescribing a more expensive ICS-formoterol inhaler compared with a SABA inhaler.

## Time for national integration of medicines optimisation with clinical outcomes?

In the UK, medicines management teams often focus entirely on reducing overall drug expenditure as a cost-saving strategy, without considering the impact on patient outcomes and the impact on primary and secondary care healthcare utilisation and costs. One of the aims of the new integrated care systems in England is to *‘improve outcomes in population health and healthcare’*^12^ and perhaps the medicines optimisation management approach should also focus on clinical cost-effectiveness^13^ and clinical outcomes^9^ than just the cost of drugs. The use of SABA and low-dose ICS regimens in mild asthma may be cheaper than ICS-formoterol therapy, but patient outcomes are worse. AIR and maintenance and reliever therapy (MART) regimens have been demonstrated to reduce SABA usage. As-needed AIR therapy results in better outcomes than the use of SABA for relief, even in those who take their prescribed maintenance ICS-based treatment *regularly*,^9^^,^^14^ while MART is more effective than using fixed-dose regular ICS/long-acting bronchodilator inhaler (LABA) regimens, which demand high levels of patient adherence, at the same and higher ICS doses.^14^

## Conclusions

We have compared low (1–2) SABA users to extreme (>12) SABA users to show that the 11+ additional SABA prescriptions increase exacerbation-related healthcare utilisation costs by £97 015 per thousand patients. This translates to an approximate £9 healthcare utilisation footprint for each SABA prescription, making SABA overprescription considerably more expensive to healthcare systems than the cost of the inhaler alone. It is apparent that UK medicines management teams that focus purely on overall drug expenditure in isolation without considering patient outcomes may unintentionally contribute to poor clinical outcomes and increased risk for patients, as well as higher overall healthcare costs across primary and secondary care. Clearly, in addition to medicines optimisation, it is important to confirm the diagnosis of asthma, as well as identification of and optimising care related to modifiable risk factors and comorbid conditions. Reduced short-acting reliever prescribing, coupled with increased inhaled corticosteroid prescribing, will reduce preventable asthma attacks, deaths, and therefore unplanned healthcare resource utilisation, as well as workload and costs.
